# Supplementary material for: Knowledge of breastfeeding practice and associated factors among fathers whose wife delivered in last one year in Gurage Zone, Ethiopia
Source: PLoS One. 2021 Jul 19;16(7):e0254824. doi: 10.1371/journal.pone.0254824 (PMC8289068; doi:10.1371/journal.pone.0254824)
Supplement: S2 File — (DOCX) [file pone.0254824.s002.docx]

ANNEX I

### Individual consent form

Greeting, my name is--------------------------------I am working as data collector in this study that assesses father’s knowledge and attitude towards breast feeding and associated factors in Gurage zone south nation nationalities and peoples region, Ethiopia. I would like to assure you that privacy will be strictly be maintained throughout. There is no need to put your name. No individual response will be reported. It is your full right to participate or refuse to participate in the study. However, your honest participation will have a great contribution. So please take a few minute to answer this question.

Would you participate in study? Yes ___________

No __________

Name and signature of the data collector who sought the consent ______________

Date of interview ________________________

### English version Questionnaire form

Socio-demographic question

| No | demographic, fathers characteristics | Answers options |  |
| --- | --- | --- | --- |
| 1 | How old are you in complete years? | ….……………. |  |
| 2 | What is your residence? | a)urban b) rural |  |
| 3 | What is your current marital status? | a) married  b) Single  c) divorced  d) widowed |  |
| 4 | What is your job? | a) student  b)government employee  c) merchant  d) House husband.  e) If other specify... .. .. .. .. ... |  |
| 5 | What is your religion? | a) Orthodox  b) Protestant  c) Catholic  d) Muslim  e) other |  |
| 6 | What is your monthly income? | ----------------------------------- |  |
| 7 | What is your ethnicity? | 1. Wolkite 2. Silt   c) Hadiya  d) Halaba  e) other |  |
| 8 | What is your educational level? | a) unable to read and write  b) read and write  c) grade complete(1-6)  d) grade complete(7-12)  e) certificate and above |  |
| 09 | How many children you have? | a) only one  b) two  c) three  d) more than three |  |

**Questions assessing knowledge**

| NO | Questions | True | False | Note sure |
| --- | --- | --- | --- | --- |
| 1 | Breast milk as the first given to infant after birth. |  |  |  |
| 2 | Water or glucose water should not be introduced to the infant to their first few days of life. |  |  |  |
| 3 | Infant formula is more beneficial to the baby than the breast milk |  |  |  |
| 4 | It is common for mothers to have insufficient milk in their breast. |  |  |  |
| 5 | Do you know if a mother who reports she feels has insufficient milk should feed with infant formula in addition to  Breastfeeding. |  |  |  |
| 6 | Mothers should stop breastfeeding if their  Baby is ill. |  |  |  |
| 7 | Breast milk alone is sufficient to provide all nourishment for infants in the first six months of life |  |  |  |
| 8 | In breast feeding session mothers should ensure that one breast is fully emptied before introducing the second breast. |  |  |  |
| 9 | Exclusive breast feeding protects mothers from pregnancy in the first few months’ afterbirth |  |  |  |
| 10 | A baby should be fed formula as soon as he/she born |  |  |  |
| 11 | Semi-solid or soft foods should not be introduced to the infant before six months |  |  |  |
| 12 | Breast feeding should be stopped the moment the baby is introduced to semi-solid or soft foods |  |  |  |

**Questions that contain determinants of knowledge**.

| NO | Questions | Yes | No |
| --- | --- | --- | --- |
| 1 | What is your educational a) grade >=9  b) grade <9 |  |  |
| 2 | Do you visit health facility |  |  |
| 3 | Does your wife positively you in breast feeding |  |  |
| 4 | Do you have TV/radio in home |  |  |

**Section B assessment of attitude**

| NO | Questions | Strongly agree | Agree | Neutral | Not agree | Not strongly agree |
| --- | --- | --- | --- | --- | --- | --- |
| **1** | The nutritional benefits of breast milk lasts only until the baby is weaned from breast milk. |  |  |  |  |  |
| **2** | Formula feeding is more convenient than breast feeding. |  |  |  |  |  |
| **3** | Breast feeding increases mother-infant bonding |  |  |  |  |  |
| **4** | Breast milk lacks iron |  |  |  |  |  |
| **5** | Formula-fed babies are more likely to be over feed than are breast-fed babies. |  |  |  |  |  |
| **6** | Formula feeding is the better choice if a mother plans to work outside the home |  |  |  |  |  |
| **7** | Mothers who formula-fed miss one of the great joys of motherhood |  |  |  |  |  |
| **8** | Women should not breast feed in public places such as restaurants |  |  |  |  |  |
| **9** | Fathers feel left out if a mother breast feeds |  |  |  |  |  |
| **10** | Breast milk is the ideal food for babies |  |  |  |  |  |
| **11** | Breast milk is more easily digested than formula |  |  |  |  |  |
| **12** | Formula is as healthy for an infant as breast milk |  |  |  |  |  |
| **13** | Breast milk is less expensive than formula |  |  |  |  |  |

**Thank you**
